# Assessment of indirect protection from maternal influenza immunization among non-vaccinated household family members in a randomized controlled trial in Sarlahi, Nepal

**DOI:** 10.1016/j.vaccine.2020.08.014

**Published:** 2020-10-07

**Authors:** Kira L. Newman, Laveta M. Stewart, Emily M. Scott, James M. Tielsch, Janet A. Englund, Subarna K. Khatry, Luke C. Mullany, Steven C. LeClerq, Laxman Shrestha, Jane M. Kuypers, Helen Y. Chu, Joanne Katz

**Affiliations:** a School of Medicine, University of Washington, Seattle, WA, USA; b Department of International Health, Johns Hopkins Bloomberg School of Public Health, Baltimore, MD, USA; c University of Colorado Anschutz Medical Campus, Denver, CO, USA; d Department of Global Health, Milken Institute School of Public Health, George Washington University, Washington, DC, USA; e Seattle Children's Hospital and Research Foundation, University of Washington, Seattle, WA, USA; f Nepal Nutrition Intervention Project, Sarlahi, Kathmandu, Nepal; g Tribhuvan University, Department of Pediatrics and Child Health, Institute of Medicine, Kathmandu, Nepal; h School of Medicine, University of Washington, Molecular Virology Laboratory, Seattle, WA, USA

**Keywords:** Influenza, Vaccine, Indirect effects of vaccination, Pregnancy, Nepal

## Abstract

Influenza is a significant cause of morbidity and mortality worldwide, and the World Health Organization highly recommends maternal vaccination during pregnancy. The indirect effect of maternal vaccination on other close contacts other than newborns is unknown. To evaluate this, we conducted a nested substudy between 2011 and 2012 of influenza and acute respiratory illness (ARI) among household members of pregnant women enrolled in a randomized placebo-controlled trial of antenatal influenza vaccination in the rural district of Sarlahi, Nepal. Women were assigned to receive influenza vaccination or placebo during pregnancy and then they and their household members were followed up to 6 months postpartum with weekly symptom surveillance and nasal swab collection. Swabs were tested by RT-PCR for influenza. Rates of laboratory-confirmed influenza and of ARI were compared between vaccine and placebo groups using generalized estimating equations with a Poisson link function. Overall, 1752 individuals in 520 households were eligible for inclusion. There were 82 laboratory-confirmed influenza illness episodes, for a rate of 7.0 per 100 person-years overall. Of the influenza strains able to be typed, 29 were influenza A, 40 were influenza B, and 6 were coinfections with influenza A and B. The rate did not differ significantly whether the household was in the vaccine or placebo group (rate ratio (RR) 1.37, 95% confidence interval (CI) 0.83–2.26). The rate of ARI was 28.5 per 100 person-years overall and did not differ by household group (RR 0.99, 95% CI 0.72–1.36). Influenza vaccination of pregnant women did not provide indirect protection of unvaccinated household members.

## Highlights

• Acute respiratory infections, including influenza, are common among household member in Nepal.
• Antenatal influenza vaccination does not confer indirect protection to household members.
• Challenges include low vaccine efficacy and limited population coverage.

## 1 Introduction

Influenza is a significant cause of morbidity and mortality worldwide, with an estimated 291,000–646,000 influenza-associated deaths annually [1]. In many low resource settings, including most of South Asia, the community-based burden of influenza remains poorly characterized [1]. The World Health Organization currently recommends that pregnant women be given highest priority for influenza vaccination, followed by other high-risk groups (i.e. children < 5 years old, elderly individuals, individuals with chronic medical comorbidities, and healthcare workers) [2]. Maternal vaccination is universally recommended based on the risk of influenza to pregnant women and the increased risk of negative birth outcomes [3], [4]. There is also evidence that vaccination during pregnancy confers protection to newborns through transplacental antibody transfer [5], [6]. The indirect effect of maternal vaccination on other close contacts is unknown.

Though the direct effect of influenza vaccination on protecting individuals from infection and illness is well-documented, as is the benefit of herd immunity from population-wide vaccination [7], the indirect effects of subgroup-specific vaccination campaigns are not well known, especially in low resource settings [8]. Prior research has evaluated the impact of vaccination campaigns targeting children in high-resource settings and shown that when a large proportion of children are vaccinated, it can lead to indirect benefits through herd immunity [9], [10], [11], though household-level effects are less clear [12], [13], [14]. In healthcare settings, vaccination of staff has been shown to provide indirect protection for patients [15], but the indirect effect of vaccinating non-healthcare-associated high-risk adults, such as pregnant women, has not been evaluated [16].

To assess this, we conducted a substudy nested within a randomized placebo-controlled trial of antenatal influenza vaccination in the rural district of Sarlahi, Nepal [6]. In the main trial, pregnant women were randomized to receive either influenza vaccination or placebo and then surveyed until 6 months postpartum for respiratory symptoms and tested for respiratory pathogens when symptomatic. In this substudy, women’s household members were included in prospective symptom surveillance and sample collection. The primary aim was to assess the indirect effect of maternal vaccination on influenza incidence among household members. This analysis estimates the rates of ARI and of laboratory-confirmed influenza among household members of pregnant women administered either influenza vaccine or placebo and compares them to assess for indirect protection.

## 2 Methods

### 2.1 Study population and sample collection

A household surveillance study was conducted as a nested substudy within a randomized trial of influenza vaccination during pregnancy in rural Nepal [6], [17]. For the trial, pregnant women were enrolled as early as pregnancy was confirmed using a pregnancy surveillance system of visits every 5 weeks and pregnancy tests for those who missed a period. Pregnant women were randomized to receive either trivalent inactivated influenza vaccination or placebo no earlier than 17 weeks gestation but no later than 34 weeks, and then followed until 6 months postpartum with weekly home visits to collect symptom data and nasal swabs in the event of respiratory illness. The vaccine used was commercially manufactured by Sanofi Pasteur and targeted influenza A/H1N1 California, influenza A/H3N2 Perth, and influenza B Brisbane (Victoria-like). At the time of randomization during the first year of the study, every third study mother and their family members were selected for participation in the household surveillance substudy. All household members living with the selected pregnant woman for >6 months were eligible to participate. Consenting participants were administered a baseline individual characteristics questionnaire. Substudy participants underwent weekly symptom surveillance with collection of a nasal swab if they reported respiratory illness. Nasal swabs were tested using real-time PCR for 12 respiratory viruses, including influenza A and B. An analysis of the other respiratory pathogens has been previously reported elsewhere [18].

Participants in the household surveillance substudy were enrolled between 14 April 2011 and 1 May 2012. Household members underwent surveillance, beginning within two weeks of maternal immunization or placebo administration, until 6 months postpartum. Households were defined as a group sharing a common address and cookstove. Because the infant of the vaccinated woman would potentially be protected through a different mechanism than other household members, this analysis excluded the infant illness episodes. Data were collected regarding sociodemographic factors of individuals and households at enrollment. Households were eligible for inclusion in analysis if they had at least one day of symptom surveillance. If more than one woman in the household was participating in the trial, households were excluded if the women were randomized to different study arms (i.e. vaccine and placebo). Households were also excluded if they did not include surveillance data on members other than the trial-enrolled mother and her infant.

### 2.2 Outcome definitions

Children were defined as aged 15 years old or younger. In participants 5 years of age and older, ARI was defined as self-report of fever and myalgia, cough, nasal congestion or runny nose, or sore throat for one or more days. In children under 5 years of age, ARI was defined as caregiver report of fever, cough, ear discharge, wheezing, or difficulty breathing on at least one day. An ARI episode was defined as distinct if there were at least 7 days without symptoms following the end of a prior episode.

Laboratory confirmed influenza in adults was defined as an illness episode with fever plus one or more of myalgia, cough, nasal congestion or runny nose, or sore throat with a concurrent PCR positive mid-nasal swab. For children aged 5 years and younger, laboratory confirmed influenza was an illness episode with any one of fever, cough, ear discharge, wheeze or difficulty breathing, concurrent with a PCR positive mid-nasal swab.

### 2.3 Statistical analysis

The primary outcomes were the incidence rates of ARI and laboratory-confirmed influenza cases among household members other than the woman enrolled in the trial and her infant. The primary exposure was whether trial-enrolled women in the household had received influenza vaccine or placebo. To calculate incidence rates, rate ratios (RR), and 95% confidence intervals (CI) for ARI and laboratory confirmed influenza in vaccine versus placebo groups, we used generalized estimating equations to control for clustering by household using an exchangeable correlation structure, an offset of the log time at risk (i.e. time under surveillance) and a Poisson link. All models were also adjusted for household member age category (child vs. adult). Within household, influenza transmission was estimated according to the same methods as Scott et al. [18]. The earliest case with reported respiratory symptoms prior to a virus-positive swab was identified as the index case and secondary household illness cases during the subsequent 14 days were calculated. A post-hoc analysis of the rate of influenza A infections in homes where trial-enrolled women received vaccine versus placebo was also conducted using the same modeling strategy as for the pre-specified primary outcomes. P values were considered significant at p < 0.05. Data were analyzed using SAS 9.4 (SAS Institute Inc., Cary, NC).

### 2.4 Human subjects

The institutional review boards (IRB) at the Johns Hopkins University Bloomberg School of Public Health, Cincinnati Children's Hospital, the Institute of Medicine at Tribhuvan University and the Nepal Health Research Council approved the randomized trial and substudy with deferral from University of Washington and Seattle Children’s Hospital’s IRB. The primary trial was registered under ClinicalTrials.gov NCT01034254. This study was carried out in close collaboration with our implementing partner organization, Nepal Netra Jyoti Sangh, under the auspices of the Social Welfare Council of the Government of Nepal.

## 3 Results

**Fig. 1:**
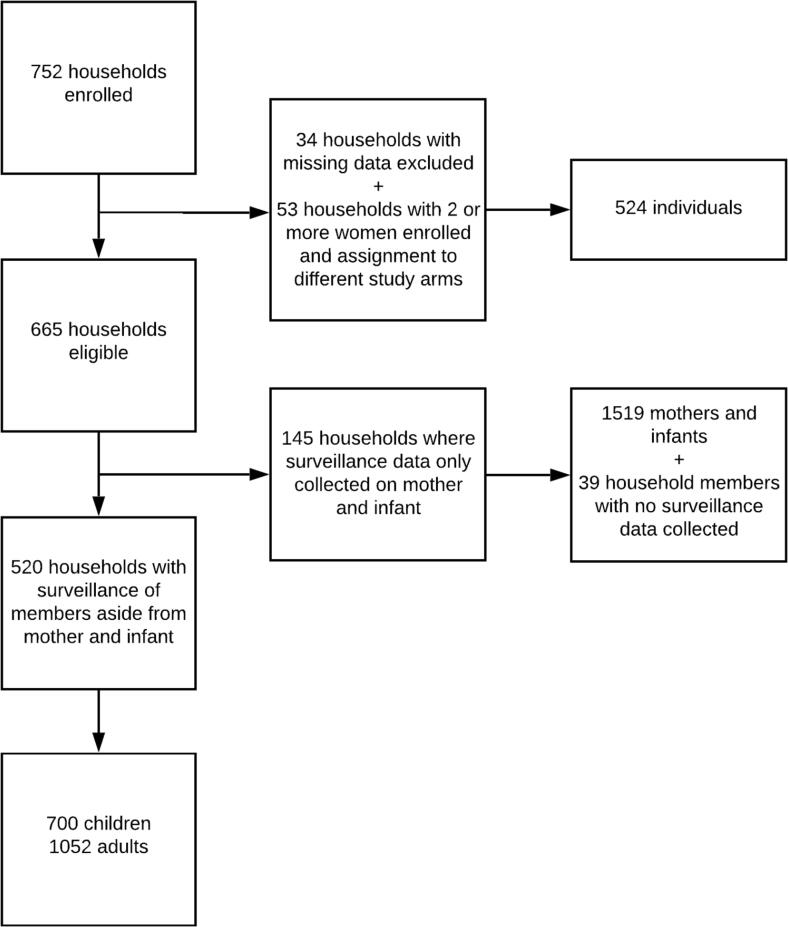
Summary of household and individual enrollment, surveillance, and inclusion in analysis of influenza in households in Sarlahi, Nepal.

A total of 752 households were enrolled in the substudy of which 520 were eligible for inclusion in the analysis, having had at least one household member other than the main trial-enrolled mother and her infant with surveillance data collected (Fig. 1). These households included 1752 individuals (excluding the main trial-enrolled mother and infant) who contributed data to the analysis. Household and individual characteristics are summarized in Table 1. Median age of included household members was 21. Of included household members, 40% were children (aged 15 years or less).

**Table 1:** Demographic and clinical characteristics of unvaccinated household members of vaccine trial participants in Sarlahi, Nepal, stratified by vaccination status of trial participant. (All n (%) unless otherwise noted).

Characteristics	Overall	Vaccine arm	Placebo arm
	n = 1752	n = 898	n = 854
Age (median, IQR)	21 (8–43)	21 (8–42)	21 (7–44)
Child (Ages 0–15 years)	700 (40.0)	353 (39.3)	347 (40.6)
Adult (Ages 16 years or more)	1052 (60.0)	545 (60.7)	507 (59.4)
Male	902 (51.5)	460 (51.2)	442 (51.8)

Household characteristics			
Electricity	1578 (90.1)	808 (90.0)	770 (90.2)
Latrine	867 (49.5)	431 (48.0)	436 (51.1)
Running water	1445 (82.5)	727 (81.0)	718 (84.1)
Crowding (>4 household members per room)	716 (40.9)	377 (42.0)	339 (39.7)
Smoker in household	839 (47.9)	443 (49.3)	396 (46.4)
Madeshi ethnicity	802 (45.8)	390 (43.4)	412 (48.2)

ARI			
1 episode	161 (9.2)	85 (9.5)	76 (8.9)
2 episodes	34 (1.9)	16 (1.8)	18 (2.1)
3 episodes	20 (1.1)	12 (1.3)	8 (0.9)
4 episodes	5 (0.3)	2 (0.2)	3 (0.4)
5 episodes	4 (0.2)	2 (0.2)	2 (0.2)

Influenza			
1 episode	68 (3.9)	39 (4.3)	29 (3.4)
2 episodes	7 (0.4)	5 (0.6)	2 (0.2)
Influenza A	35 (2.0)*	23 (2.6)**	12 (1.4)***
H1N1	13 (0.7)	9 (1.0)	4 (0.5)
H3N2	15 (0.9)	9 (1.0)	6 (0.7)
Influenza B	46 (2.6)	26 (2.9)	20 (2.3)
B/Yamagata	24 (1.4)	14 (1.6)	10 (1.2)
B/Victoria	6 (0.3)	3 (0.3)	3 (0.4)

* 6 coinfections with influenza B.

** 4 coinfections with influenza B.

*** 2 coinfections with influenza B.

### 3.1 Acute respiratory infection

**Fig. 2:**
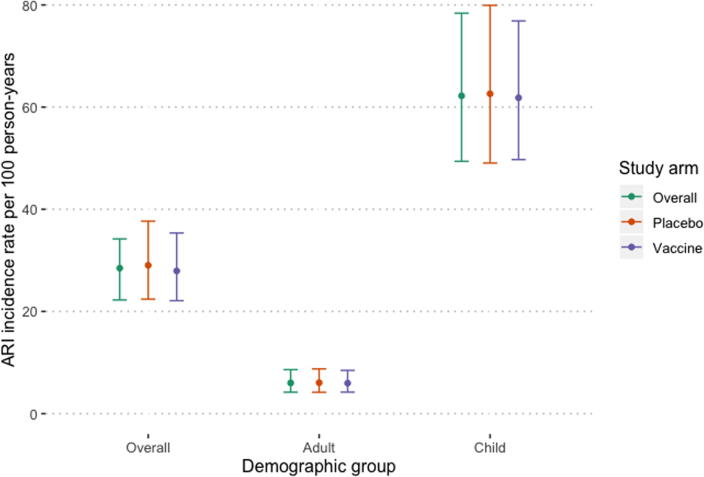
Estimated acute respiratory illness (ARI) incidence and 95% confidence intervals among unvaccinated household members of vaccine trial participants in Sarlahi, Nepal.

A total of 329 ARI episodes occurred among 224 individuals (Table 1). The overall rate of ARI among unvaccinated household members was 28.47 episodes per 100 person-years (Table 2, Fig. 2). There were significantly higher rates among children when compared to adults (p < 0.001). There was no significant difference in the rates of ARI among household members where the enrolled pregnant woman received vaccine versus those where she received placebo (RR 0.99, 95% CI 0.72–1.36).

**Table 2:** Acute respiratory illness (ARI) and laboratory-confirmed influenza infection rates per 100 person-years by group and rate ratio (RR) in unvaccinated household members of women enrolled in study of influenza vaccination in Sarlahi, Nepal.

Outcome and demographic group	Overall (rate per 100 PY and 95% CI)	Vaccine arm (rate per 100 PY and 95% CI)	Placebo arm (rate per 100 PY and 95% CI)	Crude RR* and 95% CI	Adjusted RR** and 95% CI
*ARI*					
Overall	28.47 (22.25–34.20)	27.93 (22.11–35.36)	29.03 (22.41–37.67)	1.01 (0.72–1.40)	0.99 (0.72–1.36)
Adult	6.01 (4.20–8.61)	5.97 (4.21–8.47)	6.05 (4.18–8.76)	1.11 (0.57–2.17)	–
Child	62.21 (49.39–78.38)	61.83 (49.73–76.87)	62.61 (49.05–79.92)	1.01 (0.72–1.41)	–

*Influenza*					
Overall	7.03 (4.41–8.11)	6.74 (5.04–8.86)	6.00 (3.84–7.02)	1.37 (0.83–2.26)	1.37 (0.83–2.26)
Adult	2.27 (1.22–4.22)	2.61(1.43–4.75)	1.90 (0.99–3.65)	1.19 (0.37–3.79)	–
Child	14.20 (9.88–20.43)	16.39 (11.74–22.89)	11.97 (8.00–17.93)	1.38 (0.79–2.40)	–

Abbreviations: ARI = acute respiratory illness, CI = confidence interval, PY = person years, RR = rate ratio.

* Generalized estimating equation with Poisson link and clustering by household.

** Generalized estimating equation with Poisson link and clustering by household adjusted for age category.

### 3.2 Influenza incidence

**Fig. 3:**
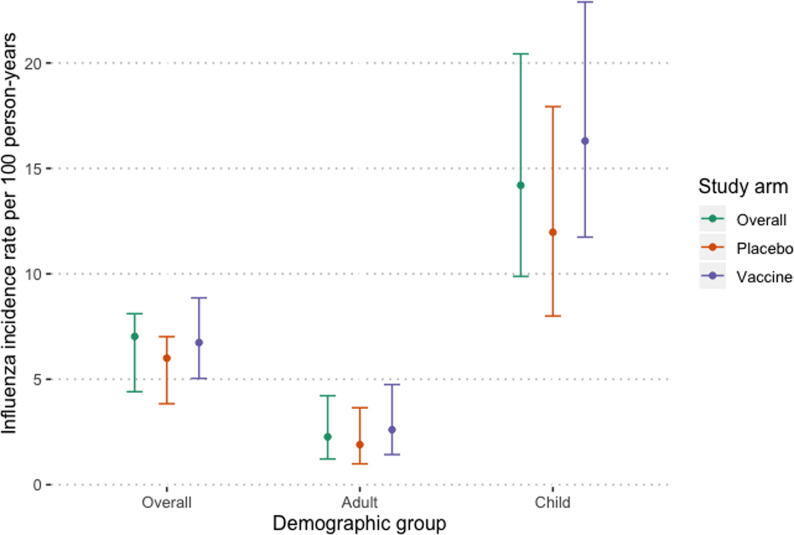
Estimated influenza incidence and 95% confidence intervals among unvaccinated household members of vaccine trial participants in Sarlahi, Nepal.

A total of 82 laboratory-confirmed influenza illness episodes occurred among 75 individuals (Table 1). There were only two cases of potential within-household transmission, in which an illness occurred in the household in the 14 days following an initial episode. Both were in households where the enrolled woman was in the vaccine arm of the main trial. The overall rate of influenza among unvaccinated household members was 7.03 cases per 100 person-years (Fig. 3). This was driven by significantly higher rates among children (p < 0.001). There was no significant difference in the rates of influenza among household members in households where the enrolled pregnant woman received vaccine versus those where she received placebo (RR 1.37, 95% CI 0.83–2.26). There were similar rates of influenza A and influenza B infections (Table 1). Most influenza B infections that were able to be typed were B/Yamagata. In the analysis of just influenza A infections, there was no significant association between the rate of infections and vaccination (RR 1.57, 95% CI 0.34–7.27).

## 4 Discussion

In this prospective longitudinal home-based active surveillance study in rural South Asia to evaluate influenza incidence and transmission, vaccination of a pregnant woman did not provide indirect protection of her unvaccinated household members. There was insufficient data to evaluate whether influenza vaccination of a pregnant household member prevented secondary transmission within the household. Our data demonstrate a significant burden of symptomatic respiratory illness among households, including laboratory-confirmed influenza.

• Lack of evidence of indirect protection may have been due to low vaccine efficacy overall due to mismatch between circulating strains and the vaccine strain, high rates of close contact with others outside the household more likely to carry influenza, and/or failure to reach a sufficient threshold of herd immunity. The trivalent inactivated vaccine used in the trial only included coverage for influenza B for Victoria-like strains, with minimal cross protection against the B/Yamagata circulating strains that were detected in the majority of participants [19]. However, we did not observe differences in indirect protection by influenza subtype, which indicates that either the vaccine had very low efficacy against circulating influenza A and B strains, that indirect protection is not achieved by vaccination of pregnant household members, or that we had an insufficient number of observed events to see an effect on the subtype level. On a global level for the 2011–2012 influenza A vaccine, the vaccine was estimated to be 65% effective against H1N1 but only 39% effective against H3N2[20]. Efficacy in the first trial cohort which corresponded to the time period of this household study was even lower, at 55% during pregnancy, 35% postpartum and 16% for infants [6]. Given the modest amount of direct protection conferred and its significant intra-seasonal waning [21], it is possible that a more effective vaccine could provide a small amount of indirect protection.

Vaccination of other household members, such as children, has shown indirect protection of household members [13], [14]. Vaccination of a child in a household during an influenza B outbreak in Hong Kong was associated with a 5% absolute risk reduction in the likelihood of influenza infection in a household member, a small effect [14]. Importantly, vaccine efficacy in that setting was 71%, which is significantly greater than the vaccine efficacy noted in our study. In contrast, an earlier study of live-attenuated vaccine in young children did not show any indirect protection of household members of vaccinated children [12].

Another reason for lack of indirect protection may have been the choice of household member in our study. Women in the region of Nepal where the study was conducted generally have more limited social networks and do not work outside the home as much as men, although many are engaged in agricultural work. In contrast, men are more likely to do jobs that take them outside the home, such as wage-earning jobs which may include regional migration [22] or agricultural responsibilities that require travel to markets and other centers to sell products [23]. This is observed across various socioeconomic, ethnic, and caste groups [23]. As a result, men may have more social contacts and opportunities for contracting influenza. Similarly, 88% of children ages 6–10 years old in rural southern Nepal attend school [22], which likely increase their risk of contracting influenza. It is possible that a policy of vaccinating adults who work outside the home or vaccinating school aged children could provide greater indirect protection to household members in this setting.

A third possible reason for the lack of indirect protection to household members by vaccinating pregnant women is that they did not make up a sufficiently large group to provide measurable herd immunity. The majority of households in the study had only one vaccinated woman and a median household size of 9 individuals [18]. While the required percentage of the population that needs to be vaccinated for herd immunity varies widely based on virus and vaccine characteristics [24], overall population rates less than 30% not susceptible are generally not sufficient for measurable indirect protection. Studies have found modest herd immunity against influenza-like illness with vaccination rates as low as 20–25% of children, with greater rates of herd immunity as coverage increases [9], [10], [11]. In our study, coverage in pregnant women represented only about 10% of the total population of enrolled households.

The large size of the trial and randomized placebo-controlled design allowed for a prospective assessment of the indirect effect of influenza vaccination of pregnant women on influenza and ARI among household members. However, there are several limitations to our study. Asymptomatic individuals were not sampled, which limits our ability to fully estimate incidence of infection. In addition, some households originally selected for the household substudy did not get surveyed as intended. In these households, adults were less likely to be surveyed than children. Among adults, males and those under 40 years old were less likely to be surveyed [18], which may be important because these individuals are may be more likely to work outside the home and travel [23]. We also surveyed more preschool-aged children than school-aged children [18]. These differential exclusions may have affected the results, including the identification of cases. Finally, we did not collect data on social networks of individuals in the study, which may have helped provide insight into community transmission.

These findings indicate that vaccination of pregnant women is insufficient to provide indirect protection to household members, particularly when vaccine strains are not well matched. Our study suggests broader vaccination campaigns may be necessary to provide protection against influenza in rural South Asia. Further research should explore whether the additional vaccination of other high-risk groups, such as young children, the elderly, and individuals with significant comorbidities, would provide indirect protection to unvaccinated individuals.

## Declaration of Competing Interest

The authors declare the following financial interests/personal relationships which may be considered as potential competing interests: ‘Kira L Newman: None. Laveta Stewart: None. Emily M Scott: None. James M Tielsch: None. Janet A. Englund: Consulting fees from Sanofi Pasteur and Meissa Vaccines; research support from AstraZeneca, GlaxoSmithKline, Novavax, and Merck. Subarna K Khatry: None. Luke C Mullany: None. Steven C LeClerq: None. Laxman Shrestha: None. Jane M Kuypers: None. Helen Y. Chu: Consulting fees from Merck and Glaxo Smith Kline, and research support from Sanofi Pasteur, Ellume, Genentech, and Cepheid outside of the submitted work. Joanne Katz: None’.
